# Motivations of physicians and nurses to practice voluntary euthanasia: a systematic review

**DOI:** 10.1186/1472-684X-13-20

**Published:** 2014-04-10

**Authors:** Lydi-Anne Vézina-Im, Mireille Lavoie, Pawel Krol, Marianne Olivier-D’Avignon

**Affiliations:** 1 Faculty of Nursing, Laval University, Québec, Canada; 2 Équipe de Recherche Michel-Sarrazin en Oncologie psychosociale et Soins palliatifs (ERMOS), Centre de recherche du CHU de Québec - Hôtel-Dieu de Québec, Québec, Canada; 3 Department of Social Sciences, Université du Québec à Chicoutimi (UQAC), Québec, Canada

**Keywords:** Euthanasia, Physician, Nurse, Motivation, Systematic review

## Abstract

**Background:**

While a number of reviews have explored the attitude of health professionals toward euthanasia, none of them documented their motivations to practice euthanasia. The objective of the present systematic review was to identify physicians’ and nurses’ motives for having the intention or for performing an act of voluntary euthanasia and compare findings from countries where the practice is legalized to those where it is not.

**Methods:**

The following databases were investigated: MEDLINE/PubMed (1950+), PsycINFO (1806+), CINAHL (1982+), EMBASE (1974+) and FRANCIS (1984+). Proquest Dissertations and Theses (1861+) was also investigated for gray literature. Additional studies were included by checking the references of the articles included in the systematic review as well as by looking at our personal collection of articles on euthanasia.

**Results:**

This paper reviews a total of 27 empirical quantitative studies out of the 1 703 articles identified at the beginning. Five studies were in countries where euthanasia is legal and 22 in countries where it is not. Seventeen studies were targeting physicians, 9 targeted nurses and 1 both health professionals. Six studies identified the motivations underlying the intention to practice euthanasia, 16 the behavior itself and 5 both intention and behavior. The category of variables most consistently associated with euthanasia is psychological variables. All categories collapsed, the four variables most frequently associated with euthanasia are past behavior, medical specialty, whether the patient is depressed and the patient’s life expectancy.

**Conclusions:**

The present review suggests that physicians and nurses are motivated to practice voluntary euthanasia especially when they are familiar with the act of euthanasia, when the patient does not have depressive symptoms and has a short life expectancy and their motivation varies according to their medical specialty. Additional studies among nurses and in countries where euthanasia is legal are needed.

## Background

In the past years, a number of reviews on the attitude of physicians and nurses toward euthanasia and physician-assisted suicide have been published. We identified four reviews focusing on physicians and six on nurses. It is worth mentioning that the reviews among both types of health professionals are fairly different. All the reviews among physicians are in a geographically defined area (United States [1,2], United Kingdom [3] or Europe [4]) and on the topic of euthanasia and physician-assisted suicide while the reviews among nurses include studies from all over the globe and only on the topic of euthanasia [5-9]. In addition, the reviews among physicians tend to estimate the percentage of physicians who support euthanasia and physician-assisted suicide [1] (or their legalization [2]) and their willingness to perform these acts [3,4], and to verify the impact of various socio-demographic characteristics such as religion, medical specialty, age and gender. Only one review among physicians documented the reasons why they are either in favor or against euthanasia and physician-assisted suicide [4].

On the other hand, the reviews among nurses mainly report nurses’ arguments for or against euthanasia [5] —including ethical principles [6,7] —or their involvement in the euthanasia process [8,9]. Only one review reported estimates of nurses’ willingness to perform euthanasia and the socio-demographic characteristics related to attitude toward euthanasia [5]. Additionally, a review focused exclusively on religion and nurses’ attitude toward euthanasia and physician-assisted suicide [10,11]. Finally, only one review was concerned with physicians’ and nurses’ attitude toward euthanasia, but the results were reported and discussed separately [1].

Notwithstanding the very useful information provided by these previous reviews, none of them compared physicians’ and nurses’ motivations to practice voluntary euthanasia and compared results from countries in which euthanasia is legal to those in which it is not. Moreover, to our knowledge, no review on euthanasia has 1) separated their findings in terms of motivation (e.g., willing to perform euthanasia) and actual performance of the behavior; 2) integrated physicians’ and nurses’ motives to perform euthanasia by classifying them according to a validated taxonomy, such as the one of Cane et al. [12]; and 3) extensively assessed the quality of each study using specific criteria. The objective of the present systematic review was thus to fill this gap in the literature by identifying physicians’ and nurses’ motives in having some intention or for performing an act of voluntary euthanasia and compare findings from countries where the practice is legalized to those where it is not.

## Methods

### Study eligibility criteria

The focus of the present systematic review was on empirical quantitative studies investigating the motivations of physicians and nurses to practice voluntary euthanasia in countries where this practice is legalized (The Netherlands, Belgium, Luxemburg, and Australia from July 1996 to March 1997) and in countries where the practice is still illegal. Studies reporting the opinion or attitude of physicians and nurses only toward the *legalization* of euthanasia were excluded. Voluntary euthanasia was defined as the act of giving a lethal injection to *deliberately* end the life of a person at the end-of-life and suffering from an incurable disease at the person’s request. Studies concerned with assisted suicide (e.g., prescription of drugs to end life), withholding and withdrawal of life-sustaining or curative treatment and non-voluntary euthanasia (i.e., not at the patient’s request) were excluded from the review. However, when studies were reporting separate results for different types of end-of-life practices (e.g., euthanasia and physician-assisted suicide), the results specifically pertaining to euthanasia were used and analyzed. When studies were reporting both quantitative and qualitative results (i.e., mixed-method studies), only the quantitative results were used and analyzed. Editorials (studies reporting the opinion of merely one person) and reviews were excluded. In addition, only studies among physicians and nurses were included in the review. Moreover, studies among students (in medicine and nursing), pharmacists, psychiatrists, social workers, directors of palliative care hospices and the general population were excluded. They also had to be concerned with the practice of euthanasia for adults suffering from an incurable disease who are near the end of life. Lastly, studies reporting euthanasia among animals, minors (pediatric), people with mental disorders (e.g., adults with dementia) and inmates were not included.

### Search strategy

The following databases were investigated: MEDLINE/PubMed (1950+), PsycINFO (1806+), CINAHL (1982+), EMBASE (1974+) and FRANCIS (1984+). Proquest Dissertations and Theses (1861+) was also investigated for gray literature (i.e., unpublished studies). No restriction was placed on the year of publication of the articles. The search was performed on April 30, 2012 and was updated to include articles until December 31, 2012 (i.e., the numbers in the flow chart include this update). In all the databases, the search terms were always related to two themes, that is euthanasia and physicians/nurses. The complete details on the search terms used in each of the database are provided in Additional file 1. In MEDLINE/PubMed, a combination of keywords and MeSH terms was used. In PsycINFO, only keywords were used because no psychological index terms corresponded to euthanasia and physicians/nurses. In CINAHL, a combination of keywords and descriptors was used. In EMBASE, a combination of keywords and Emtree was used. In FRANCIS and Proquest Dissertations and Theses, only keywords were used. Finally, the search was limited to studies published in English and French. Additional studies were also included by checking the references of the articles encompassed in the systematic review (i.e., secondary references) as well as by looking at our personal collection of articles on euthanasia.

### Study selection and data extraction

**Figure 1:**
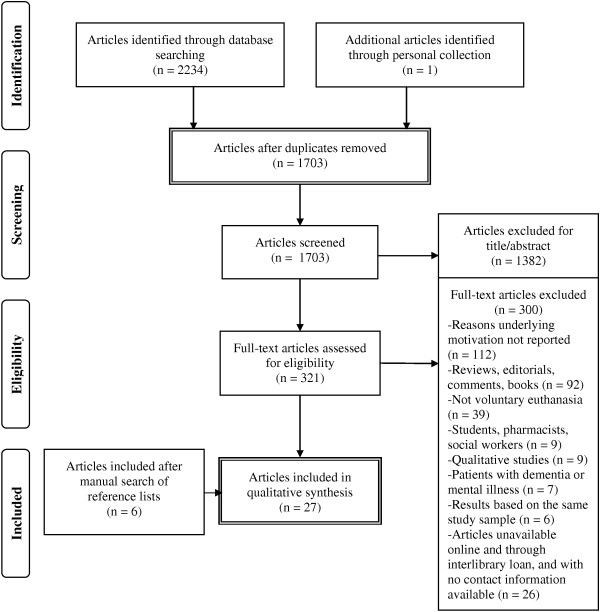
**PRISMA flow-chart [**[61]**].**

All the articles (see Figure 1) were first screened by LAVI according to their title and abstract. Clearly irrelevant articles were excluded. The remaining articles were fully retrieved (full-text) and two authors (LAVI and MOD; LAVI and PK) independently assessed them for eligibility. Disagreements were resolved by discussion, but when no consensus could be reached another author (ML) helped resolve the discrepancy.

The following data were independently extracted by LAVI and PK using a standardized data extraction form for each study: country where the study was performed, objective of the study, population of the study (physicians, nurses, both), study design (cross-sectional or longitudinal), the use of a theory, outcome measured, type of behavioral measure (objective vs. subjective, dichotomous vs. continuous), characteristics of the participants (sample size, age, gender and type of health professionals), and statistical analyses (statistical test, univariate or multivariate analyses). Physicians’ and nurses’ motives for being willing (i.e., intention) or for performing (i.e., behavior) voluntary euthanasia were classified according to an adapted version of the taxonomy of Cane et al. [12] (see Table 1 for a description). Two theoretical domains were added to the original taxonomy, moral norm and past behavior, given that a previous systematic review among health professionals had underlined their importance [13]. The previous version of the taxonomy, which was originally published by Michie et al. [14] has already been successfully applied to classify determinants of intention and/or behavior for various health behaviors [15,16], including among health professionals [13]. Again, disagreements were resolved by discussion between LAVI and PK, but when no consensus could be reached another author (ML) helped resolve the discrepancy.

**Table 1:** **Taxonomy used for classifying physicians’ and nurses’ motives for being willing or for performing euthanasia (Adapted from Cane et al.**[12]**)**

**Theoretical domain**	**Definition**	**Example applied to the field of euthanasia**
1. Knowledge	An awareness of information related to a given behavior.	Knowing the criteria for being admissible for euthanasia in countries where it is legalized.
2. Skills	An ability to perform a certain act.	Having the skills needed to perform voluntary euthanasia.
3. Social/professional role and identity	How one perceives s/he should act according to his/her social and professional identity.	Perceiving euthanasia as compatible with a caregiver’s role.
4. Beliefs about capabilities	A perceived capacity to adopt a given behavior.	Perceiving being able to perform voluntary euthanasia.
5. Beliefs about consequences	Perceived anticipated consequences of adopting the behavior.	Anticipating that euthanasia will have positive consequences for the patient, such as relieving him/her of pain.
6. Social influences	How one perceives others would react if s/he adopted a given behavior (i.e., approval or disapproval).	Perceiving that the patient’s family would approve if the physician euthanized his/her patient.
7. Emotions	Feelings arising at the thought of adopting the behavior or following behavioral adoption.	Feeling guilty or being afraid at the thought of performing euthanasia.
8. Moral norm*	How a given behavior is perceived according to one’s personal and moral values.	Perceiving euthanasia as compatible with one’s personal and moral values.
9. Past behavior*	Past experience with a given behavior.	Having already performed euthanasia in the past.

*Moral norm and past behavior were added to Cane et al.’s [12] original taxonomy.

All studies were assessed for their quality, that is according to their response rate (for surveys), their percentage of attrition at follow-up (for longitudinal studies), and their statistical analyses. For surveys (cross-sectional studies), a response rate ≥ 60% was considered ‘good’ [17] while a response rate of < 60% was considered ‘poor’ or ‘unknown’ when no information was provided. For longitudinal studies, an attrition at follow-up of ≤ 20% was considered ‘good’ [18] while an attrition of > 20% was considered ‘poor’ or ‘unknown’ when no information was provided. Lastly, the total number of variables tested in prediction models was assessed. Studies with a cases-to-predictors ratio > 15 were considered ‘good’ whereas studies with cases-to-predictors ratio ≤ 15 were rated ‘poor’, since this can be associated to problems of overfitting [19].

### Data analyses

A few studies reported results based on the same study sample. To avoid duplication of results and attributing more weight to these studies, it was decided to only use and analyze the study that would maximize the statistical power (i.e., multivariate instead of univariate analyses, bigger sample size) and allow possible meta-analysis of the results (i.e., results reported in odds ratio instead of percentages).

For each variable of a same theoretical domain, a percentage (ratio = number of time significant ÷ number of time assessed × 100) was calculated. The same formula was used to calculate results concerning socio-demographic characteristics (e.g., age, sex, religion, etc.) and patient characteristics (e.g., patient’s symptoms and suffering, patient’s life expectancy, patient’s wish, etc.) associated with euthanasia. Finally, all descriptive statistical analyses were performed using SAS version 9.3 (SAS Institute, Cary, NC, USA).

## Results

The results of the search strategy are presented in Figure 1. A total of 27 studies (n = 31 425) among independent samples were included in the present review. – In the rest of the text, the letter *k* will be used to represent the number of studies, and the letter *n* to represent the number of participants. – Given, the important heterogeneity observed between the studies, no meta-analyses of the results were performed.

### Characteristics of the studies

A summary of the studies is presented in Table 2. In the table, a statistically significant association between a variable and euthanasia is represented by a plus sign (+), a negative association by a minus sign (-), no association by a zero (0) and an unknown association by plus and minus signs (+/-). Of the 27 studies included in the systematic review, 22 studies (n = 20 692) were conducted in countries where euthanasia is not legal (United States: 10, Australia: 7, Canada: 2, Denmark: 2, Israel: 2, Sweden: 2, China: 1, Finland: 1, Germany: 1, Italy: 1, South Africa: 1, Switzerland: 1, Turkey: 1) [20-41] and 5 studies (n = 10 733) in countries where euthanasia is legal (Belgium: 3, The Netherlands: 2) [42-46]. Six studies (n = 17 434) identified the motivations underlying the intention to practice euthanasia [25,27,35,37,39,42], 16 studies (n = 12 316) the behavior itself [20-23,26,28,29,31,33,34,40],[41,43-46] and 5 studies (n = 1675) both intention and behavior [24,30,32,36,38]. Seventeen studies (n = 24 693) were conducted among physicians [21,24-29,32,34,35,37,39,40],[43-46] compared to only 9 studies (n = 6535) among nurses [20,22,23,30,31,33,38,41],[42] and 1 study (n = 197) among both types of health professionals [36]. All studies were cross-sectional (surveys) and only 2 were based on a theory. The study of Doukas et al. [24] was based on Fishbein and Ajzen’s Theory of Reasoned Action [47] and the study of Richardson [38] was based on Kohlberg’s model of moral reasoning development [48,49].

**Table 2:** Summary of studies predicting euthanasia or motivation to perform euthanasia

**Reference**	**Country**	**Outcome**	**Sample**	**Theory used**	**Response rate**	**Main results:**
						**Positive association (+)**
						**Negative association (-)**
						**No association (0)**
						**Association unknown (+/-)**
Asch & DeKay [20]	United States	Behavior	1 139 critical care nurses:	N/A	73%	• Euthanasia and PAS are unethical (-)
			Age: 38.5 (8.7)			• Passive euthanasia is unethical (-)
			5.1% male			• Working in cardiac care unit (-)
						• Ever asked to engage in euthanasia (+)
Back et al. [21]	United States	Behavior	828 physicians (GPs and specialists):	N/A	57%	Reasons for not providing euthanasia:
			Age: NR			• Physicians should never perform euthanasia
			76.3% male			• The symptoms were potentially treatable
						• The duration of the patient survival was expected to be > 6 months
						• The patient was depressed
						• The degree of patient suffering did not justify the request
						• Worried about legal consequences
Davis et al. [22]	Australia, Canada, China, Finland, Israel, Sweden and United States	Behavior	168 cancer care nurses:	N/A	N/A	• Patient wish
			Age range: 19-64			• Severe suffering
			% male: NR			• Terminally ill
						• Family agree
DeKeyser Ganz & Musgrave [23]	Israel	Behavior	71 critical care nurses	N/A	N/A	Religiosity (-)
Doukas et al. [24]	United States	Behavior and intention (willing)	154 oncologists:	Belief-attitude-intention-behavior model of Fishbein	61.6%	Behavior:
			Age: 49			• University-based oncologists have administered (+)
			83% male			Intention:
						• University-based oncologists willing (+)
						• Religion (+/-)
						• Global attitude scale (+)
						• Philosophical scale (+)
						• Alternative attitude scale (+)
Essinger [25]	United States	Intention (willingness)	365 physicians (GPs and specialists):	N/A	34%	• Deliberate administration of an overdose is never ethically justified
			Age: 48.7			
			84.7% male			• Euthanasia is inconsistent with the physician’s role to relieve pain and suffering (-)
						• Religion (-)
Folker et al. [26]	Denmark	Behavior	314 physicians (21% GPs):	N/A	64%	• Euthanasia is ethically acceptable
			Median age: 47			• Euthanasia would make me feel uncomfortable
			69% male			• Euthanasia is incompatible with my role as a physician
Inghelbrecht et al. [42]	Belgium (Flanders)	Intention (never prepared to administer lethal drugs)	3321 nurses:	N/A	62.5%	• Sex: women vs. men (+)
			77% older than 36 years			• Education: baccalaureate vs. diploma (-)
			12.4% male			Master vs. diploma (-)
						• Religion: Catholic vs. non-religious (+)
						Protestant vs. non-religious (+)
						Other religion vs. non-religious (+)
						• Work setting: home care vs. other (+)
						• Experiences with end-of-life decisions with 3 or more patients: yes vs. no (-)
Kinsella & Verhoef [27]	Canada	Intention (willingness to practice euthanasia if it were legalized)	1391 physicians (GPs and specialists):	N/A	69%	• Sex (+)
			51% over the age of 40 years			• Religious affiliation and activity (+)
			78% male			• Country of graduation (+)
Kohart [28]	United States	Behavior	93 physicians (GPs and specialists):	N/A	42.1%	• Relieve patient pain
			Age: 47			• Patient’s desire to die
			95.7% male			• Reallocate resources
						• Relieve family concern
Kuhse & Singer [29]	Australia	Behavior	869 physicians (GPs and specialists):	N/A	46%	• Euthanasia is not the doctor’s role
			Age range: < 30 to > 60 years			• Euthanasia was the right thing
			78.5% male			• Respecting the patient’s wish
						• It is right for a doctor to take active steps to bring about the death of a patient who has requested the doctor to do this
Kuhse & Singer [30]	Australia	Behavior and intention (willingness)	943 nurses:	N/A	49%	Behavior:
			40% of respondents are in their 30s			• Euthanasia was the right thing
			6% male			• Patient request
						• Discussion with the family
						• Age
						• Religion
						Intention:
						• Age
Kunene & Zungu [31]	South Africa	Behavior	26 nurses:	N/A	100%	12% would administer a lethal dose of a drug in order to relieve suffering
			Age: NR			
			8% male			
Maitra et al. [32]	Germany	Behavior and intention (willingness)	233 GPs:	N/A	48%	Behavior:
			Age: 51			• Euthanasia was right in a moral sense
			68% male			• Have received requests for euthanasia in the past (+)
Matzo [33]	United States	Behavior	441 oncology nurses:	N/A	74%	• Being married (0)
			Age: 42.0 (8.5)			• Being Jewish (0)
			2% male			• Being Catholic (0)
						• Income (0)
						• Race (0)
						• Age (0)
						• Religiosity (0)
						• Gender (0)
						• Highest degree (0)
						• Years since graduation (0)
						• Catholic religiosity (0)
						• Jewish religiosity (0)
Meeusen et al. [43]	Belgium	Behavior	205 GPs:	N/A	91.9%	Reasons for granting a patient’s request:
			Age: NR			• Explicit & repeated request from patient
			% male: NR			• Written request
						Reasons for not granting a patient’s request:
						• Patient’s wish was not explicit & repeated
						• Patient’s suffering was not unbearable & persistent
Meier et al. [34]	United States	Behavior	379 physicians:	N/A	63%	• Patient depressed at the time of request (-)
			Age: NR			• Patient in severe discomfort other than pain (+)
			% male: NR			• Patient life expectancy < 1 month (+)
Obstein et al. [44]	The Netherlands	Behavior	30 physicians:	N/A	100%	• Positive experience with euthanasia
			Age: 49.3			• No regrets after performing euthanasia
			86.7% male			• Euthanasia is part of the role of a physician
						• Euthanasia challenges personal morals
Onwuteaka-Philipsen et al. [35]	Australia, Belgium, Denmark, Italy, The Netherlands, Sweden and Switzerland (before 2002)	Intention (willingness to perform end-of-life decisions)	10 139 physicians (GPs and specialists):	N/A	57.1% (overall)	• Request of patient with decisional capacity (+)
			Age: NR			• Advance directive of subcomatose patient (+)
			% male: NR			• Request of family of patient with decisional capacity (-)
						• Subcomatose patient, request of the family (+)
						• Subcomatose patient, own initiative of physician (+)
						• Life expectancy < 2 weeks (+)
						• Uncontrollable pain (+)
						• Religious, important for professional attitude (-)
Onwuteaka-Philipsen et al. [45]	The Netherlands	Behavior	6263 physicians (GPs and specialists):	N/A	74%	Reasons for granting requests:
			Age: NR			• Wish of the patient
			% male: NR			• No prospect of improvement
						• No more options for treatment
						• Loss of dignity
Oz [36]	Turkey	Behavior and intention (willingness)	113 nurses:	N/A	Nurses: 39% Physicians: 31.8%	Nurses’ willingness to participate in legal euthanasia:
			Age: 78% between 20-30			• Age (0)
			0% male			Physicians’ willingness to participate in legal euthanasia:
			84 physicians:			• Age: 20-30 vs. 31+ (+)
			Age: 65.5% between 20-30			Nurses’ reasons for wanting to make their patient’s death easy according to years of experience:
			79.8% male			
						• Pain and depression: 7+ years vs. 1-6 years (+)
						Physicians’ reasons for wanting to make their patient’s death easy according to years of experience:
						• Pain and depression: 1-6 years vs. 7+ years: (+)
						• Insufficient support: 7+ years vs. 1-6 years (+)
Parker et al. [37]	Australia	Intention (willingness)	1478 physicians (GPs and specialists):	N/A	53%	Case 1: competent patient, life expectancy < 2 weeks: Anesthetists vs. palliative care specialists and oncologists (+)
			> 70% aged 40 or more			
						Case 2: competent patient, life expectancy > 3 months: Anesthetists vs. palliative care specialists and oncologists (+)
			78% male			Case 3: incompetent patient, life expectancy < 2 weeks: Anesthetists vs. palliative care specialists and geriatricians (+)
						Case 4: incompetent patient, life expectancy > 3 months: Anesthetists vs. palliative care specialists and geriatricians (+)
Richardson [38]	United States	Behavior and intention (attitude)	148 oncology nurses:	Kohlberg’s model of moral reasoning development	74%	Behavior:
			Age: NR			• Religious attitude to euthanasia (-)
			% male: NR			
Shapiro et al. [39]	United States	Intention (willingness)	740 physicians (GPs and specialists):	N/A	33%	Willingness to perform euthanasia:
			Age: 55.1% between 35-60			• Family/general practice vs. other specialty or internal medicine (+)
			84% male			• Christian fundamentalists vs other religions (Protestant, other) (-)
						• Catholic vs. other religions (Protestant, other) (-)
						• Jewish vs. other religions (Protestant, other) (+)
						• Specified no religion vs. other religions (Protestant, other) (+)
						Willingness to perform euthanasia if it were legalized:
						• Family/general practice vs. other specialty or internal medicine (+)
						• Christian fundamentalist vs. other religions not in analysis (-)
						• Catholic vs. other religions not in this analysis (Protestant, other), and for uncertain outcome (Christian fundamentalist, Jewish) (-)
						• Jewish vs. other religions not in this analysis (Protestant, other) (+)
						• Specified no religion vs. other religions not in analysis (+)
Smets et al. [46]	Belgium	Behavior	914 physicians (GPs and specialists):	N/A	34%	Religious affiliation/philosophy of life:
			Age: 45.1% between 51-65			• Roman Catholic/strong practicing vs. not religious (-)
			63.5% male			• Roman Catholic/moderately practicing vs. not religious (-)
						• Roman Catholic/not practicing vs. not religious (-)
						• Religious, but no specific denomination vs. not religious (-)
						Specialty:
						• Specialist vs. general practitioner (+)
						Age (years):
						• 36-50 vs. < 35 (+)
						• 51-65 vs. < 35 (+)
						• > 65 vs. < 35 (+)
						Training in palliative care: yes vs. no (+)
						Number of terminal patients cared for in the last 12 months: • 1-9 vs. 0 (+)
						• ≥ 10 vs. 0 (+)
Stevens & Hassan [40]	Australia	Behavior	298 physicians:	N/A	68%	Strong association between taking active steps and belief that such action was ‘right’
			Age: NR			Reasons why they felt they had done the ‘right’ thing:
			% male: NR			• This action had relieved pain, suffering and distress experienced by the patient
						• The patient was near death
						• The situation was hopeless
						• The patient had no prospect of a meaningful or independent existence
						• Acted on orders
Stevens & Hassan [41]	Australia	Behavior	278 nurses:	N/A	55%	Sex: male vs. female
			Age range: 20-59			
			6.5% male			

Note. *GP*s general practitioners, *N*/*A* not applicable or not available, *NR* not reported, *PAS* physician-assisted suicide, *vs*. versus.

Among the 16 studies that measured behavior, 14 used a self-reported measure of euthanasia [20-24,29,30,32-34,40,41,44,46] and 13 used a dichotomous score (yes/no) [20,21,23,24,29,30,32,34],[40,41,43,45,46]. The mean response rate was 60.6% (range: 31.8-100%). Only 8 studies verified whether respondents differed from non-respondents in terms of socio-demographic characteristics [21,26,27,33,35,39,42,46]. Ten studies did not report any statistical test [22,26,28-31,40,43-45], 9 studies reported descriptive analyses (e.g., χ^2^) [21,23-25,27,36-38,41] and 8 studies reported predictive analyses (e.g., logistic or linear regression) [20,32-35,39,42,46]. Among the studies reporting statistical tests, 10 had univariate analyses [21,23-25,27,36-38,41,42] and 7 had multivariate analyses [20,32-35,39,46] with a mean number of 8 variables tested (range: 3-16). Finally, the quality assessment of each study is reported in Table 3.

**Table 3:** Quality assessment of the studies

**Reference**	**Response rate ≥ 60%**	**Verified whether respondents differed from non-respondents**	**Cases-to-predictors ratio > 15 for multivariate analyses**
Asch & DeKay [20]	√		√
Back et al. [21]		√	N/A
Davis et al. [22]	NR		NR
DeKeyser Ganz & Musgrave [23]	NR		N/A
Doukas et al. [24]	√		N/A
Essinger [25]			N/A
Folker et al. [26]	√	√	NR
Inghelbrecht et al. [42]	√	√	N/A
Kinsella & Verhoef [27]	√	√	N/A
Kohart [28]			NR
Kuhse & Singer [29]			NR
Kuhse & Singer [30]			NR
Kunene & Zungu [31]	√		NR
Maitra et al. [32]			√
Matzo [33]	√	√	√
Meeusen et al. [43]	√		NR
Meier et al. [34]	√		√
Obstein et al. [44]	√		NR
Onwuteaka-Philipsen et al. [35]		√	√
Onwuteaka-Philipsen et al. [45]	√		NR
Oz [36]			N/A
Parker et al. [37]			N/A
Richardson [38]	√		N/A
Shapiro et al. [39]		√	√
Smets et al. [46]		√	√
Stevens & Hassan [40]	√		NR
Stevens & Hassan [41]			N/A

Note. *N*/*A* not applicable, *NR* not reported.

√ indicates a yes.

### Characteristics of the participants

Only 8 studies provided information on the mean age of their participants [20,23-25,28,32,33,44]. The pooled mean age of the participants in those 8 studies was 45.28 ± 5.45 years (range: 36.7-51 years). Nineteen studies indicated the percentage of male participants in their sample [20,21,23-33,37,39,41,42,44],[46]. A bit more than half (52.39%) of the samples were composed of male respondents. The typical sample was thus composed of middle-aged physicians with slightly more than half being male participants.

### Most consistent variables associated with behavior and/or intention

Due to the small number of studies included in the review, the variables associated with behavior and intention are reported jointly in Table 4. Types of variables that are the most frequently assessed are the socio-demographic characteristics of health professionals (assessed 68 times for 12 variables = 5.67), followed by psychological variables (assessed 27 times for 6 variables = 4.50) and finally, patient characteristics (assessed 35 times for 8 variables = 4.38). Categories of variables that are the most consistently and significantly associated with euthanasia are psychological variables (37.0%), followed by socio-demographic variables (33.8%) and patient variables (i.e., variables related to the patient’s condition) (31.4%). Finally, all categories collapsed, the four most consistent variables associated with euthanasia are past behavior (100%), health professionals’ medical specialty or work setting (66.6%), whether the patient is depressed (66.6%) and the patient’s life expectancy (60.0%).

**Table 4:** **Variables measured and associated with behavior and**/**or intention for physicians and nurses (k** = **27)**

**Variables measured**	**Number of time**		**Ratio**
	**Assessed**	**Significant (*p*** **< 0.05)**	**(%)**
Psychological variables*			
Past behavior	3	3	100%
Beliefs about consequences	5	2	40.0%
Social/professional role and identity	6	2	33.3%
Beliefs about capabilities	3	1	33.3%
Moral norm	9	2	22.2%
Emotions	1	0	N/A
Total	27	10	37.0%
***Socio***-demographic variables**			
Medical specialty, unit and work setting	9	6	66.6%
Religion	17	7	41.2%
Number of terminal patients	3	1	33.3%
Gender	10	3	30.0%
Level of education	4	1	25.0%
Years of work experience	5	1	20.0%
Age	12	2	16.6%
Marital status	3	0	0%
Place of birth	2	1	N/A
Had training in palliative care	1	1	N/A
Income	1	0	N/A
Ethnicity	1	0	N/A
Total	68	23	33.8%
Patient variables**			
Patient depressed	3	2	66.6%
Patient’s life expectancy	5	3	60.0%
Patient’s symptoms and suffering	10	4	40.0%
Family agreement	4	1	25.0%
Patient’s wish	7	1	14.3%
Condition with no prospect of improvement	4	0	0%
Loss of dignity	1	0	N/A
To reallocate resources	1	0	N/A
Total	35	11	31.4%

Note. *N*/*A* not computed because it was not assessed at least three times.

*Categories based on the taxonomy of Cane et al. [12].

**Categories based on the variables identified in the studies included.

### Most consistent variables associated with behavior and/or intention according to health profession

The variables associated with euthanasia according to health profession are presented in Table 5. Our data suggests that among physicians, the two types of variables that are the most frequently assessed are socio-demographic characteristics (assessed 33 times for 9 variables = 3.67) and psychological variables (assessed 22 times for 6 variables = 3.67), closely followed by patient variables (assessed 26 times for 8 variables = 3.25). Categories of variables that are the most consistently and significantly associated with euthanasia among physicians are socio-demographic variables (42.4%), followed closely by patient variables (42.3%) and psychological variables (31.8%). All categories collapsed, the four most consistent variables associated with euthanasia among physicians are the patient’s life expectancy (75.0%), physicians’ medical specialty, unit or work setting (66.6%), the patient’s symptoms and suffering (57.1%) and physicians’ religion (55.5%). Unfortunately, due to the small number of studies among nurses (k = 9), individual ratios for the socio-demographic variables only could be computed. Although few studies (k = 4) assessed psychological variables among nurses, it seems to be the category most consistently associated with euthanasia (75%), followed by socio-demographic variables (21.2%). For nurses, patient variables (0%) did not seem much related to euthanasia. The two most consistent socio-demographic variables associated with euthanasia among nurses are their medical specialty or work setting (66.6%) and gender (50%).

**Table 5:** **Variables measured and associated with behavior and**/**or intention according to health profession**

**Variables measured**	**Number of time**		**Ratio**
	**Assessed**	**Significant (*p*** **< 0.05)**	**(%)**
Physicians (k = 17)			
***Psychological variables***			
Beliefs about consequences	5	2	40.0%
Social/professional role and identity	6	2	33.3%
Moral norm	7	1	14.3%
Beliefs about capabilities	2	1	N/A
Past behavior	1	1	N/A
Emotions	1	0	N/A
Total	22	7	31.8%
***Socio***-***demographic variables***			
Medical specialty, unit and work setting	6	4	66.6%
Religion	9	5	55.5%
Number of terminal patients	3	1	33.3%
Age	5	1	20.0%
Gender	6	1	16.6%
Had training in palliative care	1	1	N/A
Place of birth	1	1	N/A
Years of experience	1	0	N/A
Marital status	1	0	N/A
Total	33	14	42.4%
***Patient variables***			
Patient’s life expectancy	4	3	75.0%
Patient’s symptoms and suffering	7	4	57.1%
Patient’s wish	5	1	20.0%
Condition with no prospect of improvement	4	0	0%
Patient depressed	2	2	N/A
Family agreement	2	1	N/A
Loss of dignity	1	0	N/A
To reallocate resources	1	0	N/A
Total	26	11	42.3%
Nurses (k = 9)			
***Psychological variables***			
Past behavior	2	2	N/A
Moral norm	2	1	N/A
Total	4	3	75%
***Socio***-***demographic variables***			
Medical specialty, unit and work setting	3	2	66.6%
Gender	4	2	50.0%
Religion	8	2	25.0%
Level of education	4	1	25.0%
Age	6	0	0%
Years of experience	3	0	0%
Marital status	2	0	N/A
Place of birth	1	0	N/A
Income	1	0	N/A
Ethnicity	1	0	N/A
Total	33	7	21.2%
***Patient variables***			
Patient’s symptoms and suffering	2	0	N/A
Patient’s wish	2	0	N/A
Family agreement	2	0	N/A
Patient’s life expectancy	1	0	N/A
Total	7	0	0%

Note. *N*/*A* not computed because it was not assessed at least three times.

### Most consistent variables associated with behavior and/or intention according to legal status of euthanasia

The variables associated with euthanasia according to the legal status of euthanasia (i.e., country where euthanasia is legalized or not) are presented in Table 6. In countries where euthanasia is *not* legal, types of variables that are the most frequently assessed are the socio-demographic characteristics (assessed 57 times for 11 variables = 5.18), followed by patient variables (assessed 29 times for 7 variables = 4.14), and psychological variables (assessed 22 times for 6 variables = 3.67). The categories of variables that are the most consistently and significantly associated with euthanasia in countries where euthanasia is *not* legal are the psychological variables (40.9%), followed by the patient variables (37.9%) and the socio-demographic variables (24.6%). All categories collapsed, the five most consistent variables associated with euthanasia in countries where the act is *not* legal are equally beliefs about consequences—or attitude— (66.6%) and whether the patient is depressed (66.6%), followed by the patient’s life expectancy (60.0%) and health professionals’ medical specialty or work setting (57.1%). Unfortunately, due to the small number of studies in countries where euthanasia is legal (k = 5), no individual ratios could be computed. Though it can be said that the category of variables that seems to be most consistently associated with euthanasia in countries where the act is legal is by far socio-demographic variables (90.0%), followed by psychological variables (20.0%). Similarly to studies among nurses, it appears that patient variables (0%) are unrelated to euthanasia in countries where the act is legal.

**Table 6:** **Variables measured and associated with behavior and**/**or intention according to legal status of euthanasia**

**Variables measured**	**Number of time**		**Ratio**
	**Assessed**	**Significant (*p*** **< 0.05)**	**(%)**
Countries were euthanasia is not legal (k = 22)			
***Psychological variables***			
Beliefs about consequences	3	2	66.6%
Social/professional role and identity	5	2	40.0%
Beliefs about capabilities	3	1	33.3%
Moral norm	8	2	25.0%
Past behavior	2	2	N/A
Emotions	1	0	N/A
Total	22	9	40.9%
***Socio-demographic variables***			
Medical specialty, unit and work setting	7	4	57.1%
Religion	15	5	33.3%
Years of experience	4	1	25.0%
Gender	9	2	22.2%
Age	10	1	10.0%
Level of education	3	0	0%
Marital status	3	0	0%
Place of birth	2	1	N/A
Number of terminal patients	2	0	N/A
Income	1	0	N/A
Ethnicity	1	0	N/A
Total	57	14	24.6%
***Patient variables***			
Patient depressed	3	2	66.6%
Patient’s life expectancy	5	3	60.0%
Patient’s symptoms and suffering	9	4	44.4%
Family agreement	4	1	25.0%
Patient’s wish	5	1	20.0%
Condition with no prospect of improvement	2	0	N/A
To reallocate resources	1	0	N/A
Total	29	11	37.9%
Countries where euthanasia is legal (k = 5)			
***Psychological variables***			
Beliefs about consequences	2	0	N/A
Past behavior	1	1	N/A
Social/professional role and identity	1	0	N/A
Moral norm	1	0	N/A
Total	5	1	20.0%
***Socio***-***demographic variables***			
Medical specialty, unit and work setting	2	2	N/A
Religion	2	2	N/A
Age	2	1	N/A
Gender	1	1	N/A
Had training in palliative care	1	1	N/A
Number of terminal patients	1	1	N/A
Level of education	1	1	N/A
Total	10	9	90.0%
***Patient variables***			
Patient’s wish	2	0	N/A
Condition with no prospect of improvement	2	0	N/A
Patient’s symptoms and suffering	1	0	N/A
Loss of dignity	1	0	N/A
Total	6	0	0%

Note. *N*/*A* not computed because it was not assessed at least three times.

## Discussion

According to our review, the first studies on physicians’ and nurses’ motivation to practice voluntary euthanasia were published in the 1970s. Still, more than 4 decades later, the number of scientific papers available in the literature is low (k = 27). When we consider our flowchart (see Figure 1), it seems mostly due to the fact that few studies investigated the reasons underlying health professionals’ motivation to perform euthanasia and a number of studies are opinion or comment articles (i.e., editorials).

### Psychological variables associated with euthanasia

To our knowledge, this is the first systematic review to use an adapted version of the taxonomy of Cane et al. [12] to classify physicians’ and nurses’ psychological motives to practice euthanasia. In fact, the present review revealed that psychological variables are the category of variables most consistently associated with euthanasia. The single most important variable associated with euthanasia was past behavior. It was the only variable that had a 100% assessed-significant ratio. Our analysis then suggests that, when health professionals are familiar with practicing euthanasia, they seem more motivated to perform an act of euthanasia. This result is in agreement with the scientific literature in the field of health psychology, where past behavior or habit is often the main determinant of intention or behavior adoption [50]. In addition, this finding is also in agreement with some of the statements of the Theory of Interpersonal Behavior of Triandis [51] —a theory offering explanations to identify the determinants of ethical behaviors, such as euthanasia—which states that the frequency with which a behavior is performed (i.e., strength of habit) predicts behavior adoption, alongside intention. More precisely, according to Triandis’s theory, initial behavior adoption is mostly governed by intention because the person has no experience with the behavior. Eventually, the more one has adopted a behavior in the past (or the more experience one has with a behavior), the more the behavior becomes governed by habit—the person does not have to give it as much thought as s/he initially did. In other words, it is said that the behavior is more automatic. In the case of euthanasia though, where it is highly unlikely that it becomes an habitual behavior, it is possible that once health professionals have overcome potential barriers to performing this act, such as feelings of fear and guilt or respect for sanctity of human life, they become more motivated to do it again. They might also feel more at ease but also more confident in practicing euthanasia. In fact, according to the Theory of Interpersonal Behavior, habit and personal abilities are closely related.

Other psychological variables less often associated with euthanasia were beliefs about consequences, social/professional role and identity, beliefs about capabilities and moral norm. None of these variables were significantly related to euthanasia in more than half of the studies that assessed them (i.e., all ratios < 50%). This again stresses the importance of past experience over perceiving positive consequences of euthanasia, feeling that it is congruent with physicians’ and nurses’ role, perceiving that one has the ability to perform this act and believing that euthanasia is compatible with physicians’ and nurses’ personal and moral values. In other words, for now, those variables do not contribute to explain physicians’ or nurses’ motivation to practice euthanasia.

### Socio-demographic variables associated with euthanasia

Socio-demographic variables were the second type of variables most consistently associated with euthanasia. The main one was health professionals’ medical specialty or work setting. – In the rest of the text, medical specialty will refer to physicians, while work setting will refer to nurses. –This result corroborates those of a previous review of attitudes among US physicians [1]. This indicates that health professionals will be favorable (i.e., have positive attitudes) and be motivated to perform euthanasia depending on their medical specialty or work setting. A possible explanation for this observation is that in certain medical specialty and work setting, exposure to suffering, such as patients with advanced chronic diseases, can be more common than in other specialties or settings and thus influence health professionals’ intention to practice euthanasia.

Surprisingly, religion, while being the single variable most frequently assessed, was not significantly related to euthanasia in more than half of the studies. This contradicts previous reviews that clearly identified religion as an important factor in physicians’ and nurses’ attitude towards euthanasia [1-5,8-10]. Yet, a previous review had underlined important methodological flaws in the operationalization of religion in surveys, with questions often being too vague to assess the impact of religion on nurses’ attitudes toward euthanasia [11]. Moreover, it also seems that most studies use a simple checklist to determine religious affiliation, which might to be too simplistic to fully capture its influence on euthanasia [52].

The other socio-demographic variables were the number of terminal patients, gender, level of education, years of work experience, and age. The higher the number of terminal patients physicians cared for in the last 12 months, the more chances they had to perform euthanasia [46]. Male health professionals were more willing to practice euthanasia compared to their female counterparts [27,41,42]. Nurses with higher levels of education, such as baccalaureate and master’s degrees, were more inclined to administer lethal drugs compared to those with a diploma or an associate degree [42]. Nurses with more than 6 years of work experience were more willing to practice euthanasia to relieve the patient’s pain and depression, while on the opposite, physicians with more than 6 years of experience were less willing to adopt this behavior [36]. Comparatively to a previous review among European physicians [4], the results concerning age were more contradictory. According to the study of Oz [36], more physicians aged between 20 and 30 years were willing to participate in legal euthanasia compared to their counterparts who were more than 31 years old. On the contrary, the study of Smets et al. [46] indicated that the older the physicians were, the more likely they were to perform euthanasia.

### Patient variables associated with euthanasia

Variables related to the patient’s condition were the least consistently associated with euthanasia, even though it was the category of variables most frequently assessed. Physicians were motivated to practice euthanasia in the case of patients *not* depressed [21,34], with a short life expectancy (less than 2 weeks [35] or less than 1 month [34]), with severe symptoms and suffering [21,34,35], when patients’ family agreed with the decision [35] and when it was patients’ wish [35]. Patient variables were unrelated to euthanasia among nurses and in countries where the act is legal.

### Variables associated with euthanasia according to health profession

The present systematic review revealed that there is almost twice the number of studies among physicians compared to those among nurses. In fact, a low number of studies among nurses precluded the computation of many individual ratios. While the results among nurses need to be interpreted cautiously given the low number of studies, it seems that physicians and nurses differ in their motives for performing euthanasia. For physicians, the two most important categories of variables associated with euthanasia are socio-demographic variables and patient variables while for nurses, psychological variables seem to be the most important type of variables related to euthanasia.

Still, a common important socio-demographic variable for physicians and nurses is medical specialty or work setting. According to the study of Shapiro et al. [39], general practitioners are more willing to practice euthanasia compared to specialists; paradoxically the study of Smets et al. [46] states that specialists are more motivated to perform euthanasia compared to general practitioners. Another study, carried out by Parker et al. [37], suggests that physicians specialized in anesthesia are the most willing to give lethal drugs to hasten the death of a patient’s life. Among nurses, the study of Asch and DeKay [20] indicates that less nurses reported having performed euthanasia when they were working on a cardiac care unit. In sum, while physicians’ medical specialty and nurses’ work setting seem to contribute to their motivation to practice euthanasia, the results of the different studies can be contradictory, thus making it hard to identify exactly which medical specialties or work settings contain health professionals motivated to adopt this behavior.

In addition, religion seems to exert more influence on physicians than on nurses, while gender appears more influent for nurses than physicians. It is also interesting to note that patient variables, such as the patient’s life expectancy and his/her symptoms and suffering, are important for physicians and that this does not seem to be the case for nurses. It appears that patient’s life expectancy, symptoms and suffering and his/her wishes influence physicians’ decision to practice an act of euthanasia. A previous review among European physicians had already identified that the patient’s right to decide about his/her own life and death was a reason explaining why they were favorable to euthanasia [4]. As for nurses, this seems to indicate that those variables do not motivate their intention to practice euthanasia as much as it does for physicians. As previously mentioned, current studies suggest that nurses are mainly influenced by their work setting.

### Variables associated with euthanasia according to legal status of euthanasia

There were almost five times more studies conducted in countries where euthanasia is *not* legal compared to those in countries where the act is legalized. This might be due to the fact that euthanasia is only legalized in a few countries (The Netherlands, Belgium and Luxemburg) and that the legalization is fairly recent (2002 for The Netherlands and Belgium, and 2009 for Luxemburg). Unfortunately, the low number of studies in countries where euthanasia is legal prevented the computation of any individual ratios and thus the comparison between countries where the act is legalized and those where it is not.

### Areas where additional studies are needed and recommendations

In light of this systematic review, it appears that additional studies among nurses and in countries where euthanasia is legal (The Netherlands, Belgium and Luxemburg) are needed. Accordingly, we argue that there is a clear need to perform more studies that compare physicians’ and nurses’ motives to perform euthanasia. Also, more studies should assess psychological variables given that this category of variables is underrepresented in the literature compared to socio-demographic variables and also given its importance in health professionals’ motivation to practice euthanasia.

A few recommendations for future studies are worth mentioning. First, no studies differentiated between motivation (intention) and the behavior itself. While at first, this may seem trivial, psychosocial theories, such as the Theory of Interpersonal Behavior [51] and the Theory of Planned Behavior [53], clearly distinguish motivation from actual performance of the behavior. According to these two theories, intention might predict behavior adoption. In other words, when one develops the intention or is motivated to adopt a given behavior, he/she will usually adopt it. However, in the field of health psychology, there is a phenomenon that some authors label the ‘intention-behavior gap’ wherein people having the intention to adopt a behavior fail to act on their positive intentions for various reasons (e.g., forgetfulness, lack of time, etc.) [54]. Thus, it is generally preferable, when applicable, for studies to measure behavior and not just intention alone.

Second, few studies were based on a theory. Yet, increasingly more authors are calling for more theory-based studies, given that the use of theories presents many advantages [55-58]. For instance, theory can guide the selection of determinants to be tested as potential predictors of intention or behavior [59]. The use of theory can also facilitate replication of previous findings which is crucial to increase scientific knowledge about certain behaviors [60]. Additionally, this can provide a basis for refining and developing better theories [59].

Third, the present review did not identify any longitudinal studies. It would therefore by interesting to have studies with a longitudinal design to verify if physicians’ and nurses’ motives are stable or vary in time. A review of methodological issues in assisted suicide and euthanasia research in 2000 had already recommended conducting longitudinal studies [52].

Fourth, and most importantly, studies should assess and report the psychometric qualities of the items used in their questionnaires. This information assesses the internal validity of a study and therefore its credibility. Another methodological aspect worth improving in future studies is to verify whether respondents differed from non-respondents in terms of socio-demographic variables [52] as it assesses whether the results can be generalized to the whole population of physicians and nurses (i.e., external validity). Only a handful of studies reported this information. Finally, future studies should favor the use of more powerful statistical analyses, such as multivariate regression analyses or multivariate analysis of variance (MANOVA), and add variables that significantly differ between respondents and non-respondents as covariates in statistical models [52].

### Limitations of the systematic review

The present review has some limitations that are worth mentioning. First, the small number of studies, their variability (i.e., heterogeneity) and the lack of some statistical data in some studies prevented the computation of pooled effect sizes (meta-analysis). The low number of studies also precluded the computation of some individual ratios. Second, we were unable to fulfill our first objective, which was to separate our findings in terms of motivation (e.g., willing to perform euthanasia) and actual performance of the behavior, again due to the low number of studies. Third, a number of studies did not report the necessary statistical analyses to determine whether the motives reported were statistically significant (*p* < .05) or not. Consequently, the ratios computed could underestimate the significance of some variables. Finally, the exclusive focus on quantitative data might have contributed to a loss of information contained in qualitative studies.

## Conclusions

To our knowledge, this is the first study that compared physicians’ and nurses’ motivations to practice voluntary euthanasia, and the results from studies in which euthanasia is legal to those in which it is not. It is also the first application of the taxonomy of Cane et al. [12] to classify health professionals’ psychological variables associated with euthanasia. Other novel aspects of the review are the assessment of the quality of each study using specific criteria and the report of results by following the PRISMA statement guidelines [61]. As such, the present review contributes to improve current knowledge, to identify gaps in knowledge, and to suggest new directions for future studies investigating health professionals’ motivation to perform euthanasia.

Finally, as previously mentioned, it is surprising that so few studies have been carried out on the intention or motivations of healthcare providers to practice euthanasia considering the media attention devoted to this topic in society in general. But at the same time, this might be explained by the fact that it is a sensible topic, questioning what is morally wrong or good – and legal or not –and about a matter of life and death. It is our conviction that now is the time to give it the attention it deserves considering its potential impact on healthcare services and healthcare providers themselves.

## Competing interests

The authors declare they are no competing interests.

## Authors’ contributions

LAVI screened and codified the articles, extracted the data and wrote the first draft of the manuscript. ML had the idea to perform the systematic review, contributed to resolve discrepancies between the people responsible for the screening and codification of the articles, revised and made substantial contributions to the manuscript. PK confirmed the inclusion of the articles during screening, did the second codification of the articles and revised and approved the manuscript. MOD confirmed the inclusion of some articles during screening and revised and approved the manuscript. All authors read and approved the final manuscript.

## Authors’ information

LAVI holds a bachelor of arts in psychology and a master in community health. She is currently a research professional at the Faculty of Nursing of Laval University and a PhD student at the same university. She has experience in writing scientific papers and conducting systematic reviews and meta-analyses. ML is a registered nurse and holds a PhD in philosophy on the philosophy of palliative care. She is currently a full professor and researcher at the Faculty of Nursing of Laval University and at the Research Center of the CHU de Québec. PK is a registered nurse, holds a PhD in nursing and is an adjunct professor at the Faculty of Nursing of Laval University. MOD is a social worker, holds a PhD in social work and is an adjunct professor at the Department of Social Sciences at the Université du Québec à Chicoutimi (UQAC).

## Pre-publication history

The pre-publication history for this paper can be accessed here:

http://www.biomedcentral.com/1472-684X/13/20/prepub

## Supplementary Material

Additional file 1 Documenting the search. Click here for file
