# Supplementary material for: Motivations of physicians and nurses to practice voluntary euthanasia: a systematic review
Source: BMC Palliat Care. 2014 Apr 10;13:20. doi: 10.1186/1472-684X-13-20 (PMC4021095; doi:10.1186/1472-684X-13-20)
Supplement: Additional file 1 — Documenting the search. [file 1472-684X-13-20-S1.docx]

Additional file 1. Documenting the search

| Name of the database (range of dates) | MEDLINE/PubMed (1950+) |
| --- | --- |
| Dates of the search | May 11, 2012 (April 30, 2012) |
| Initials of the person who ran the search | LAVI |
| Search terms / MeSH | euthanasia (Title/Abstract) OR active euthanasia (MeSH major topic)  AND  physicians (MeSH terms) OR nurses (MeSH terms) |
| Limits | -Article written in English and French only  -Species: humans |
| Number of hits | 820 |

| Name of the database (range of dates) | PsycINFO (1806+) |
| --- | --- |
| Dates of the search | May 11, 2012 (April 30, 2012) |
| Initials of the person who ran the search | LAVI |
| Search terms | euthanasia (Title) OR active euthanasia (Title) |
| Limits | -Publication type: journal |
| Number of hits | 531 |

| Name of the database (range of dates) | CINAHL (1982+) |
| --- | --- |
| Dates of the search | May 11, 2012 (April 30, 2012) |
| Initials of the person who ran the search | LAVI |
| Search terms / Descriptors | euthanasia (Exact major subject heading) OR active euthanasia (Title) |
| Limits | -Article written in English and French only  -Species: humans |
| Number of hits | 303 |

| Name of the database (range of dates) | EMBASE (1974+) (April 30, 2012) |
| --- | --- |
| Dates of the search | May 11, 2012 |
| Initials of the person who ran the search | LAVI |
| Search terms / Emtree | euthanasia (major focus) OR active euthanasia (major focus)  AND  physician (major focus) OR nurse (major focus) |
| Limits | -Article written in English and French only  -Species: humans |
| Number of hits | 288 |

| Name of the database (range of dates) | Proquest Dissertation and Theses (1861+) |
| --- | --- |
| Dates of the search | May 11, 2012 (April 30, 2012) |
| Initials of the person who ran the search | LAVI |
| Search terms | euthanasia (citation and abstract) OR active euthanasia (citation and abstract)  AND  physician (citation and abstract) OR nurse (citation and abstract) |
| Limits | N/A |
| Number of hits | 86 |

| Name of the database (range of dates) | FRANCIS (1984+) (April 30, 2012) |
| --- | --- |
| Dates of the search | May 11, 2012 |
| Initials of the person who ran the search | LAVI |
| Search terms | euthanasia (keyword) OR active euthanasia (keyword)  AND  physician (keyword) OR nurse (keyword) |
| Limits | Journal articles only |
| Number of hits | 157 |
